# Proteomic and metabolomic analysis of human follicular fluid from follicles with different diameters

**DOI:** 10.3389/fcell.2026.1742437

**Published:** 2026-07-30

**Authors:** Lile Jiang, Shujun Yang, Yiwen Wang, Juanke Xie, Jiaxuan Geng, Helong Zhang, Yixuan Zhang, Qian Wang, Yixuan Chen, Xingyi Wang, Cuilian Zhang

**Affiliations:** 1 Reproductive Medical Center, People’s Hospital of Henan University, Zhengzhou, China; 2 Reproductive Medical Center, Henan Provincial People’s Hospital, Zhengzhou, China; 3 Reproductive Medical Center, People’s Hospital of Zhengzhou University, Zhengzhou, China

**Keywords:** follicular fluid, *in vitro* maturation, metabolomic, oocyte, proteomic

## Abstract

**Introduction:**

Follicular fluid forms the essential microenvironment for oocyte growth and maturation, with its molecular features tightly correlated with oocyte developmental competence. Elucidating molecular differences in follicular fluid among follicles of different diameters is crucial for deciphering the mechanisms governing follicular development.

**Methods:**

In this study, we constructed a proteome-metabolome database of human follicular fluid derived from small and large follicles using multi-omics profiling.

**Results:**

Integrated analysis identified 81 differentially abundant proteins (DAPs) and 141 differentially abundant metabolites (DAMs) between the two groups. Functional enrichment analyses (GO/KEGG) demonstrated that DAPs are predominantly involved in intercellular signaling, apoptosis, and substance metabolism, while DAMs mainly participate in the metabolic and biosynthetic processes of various substances. Focusing on the apoptosis pathway --a key regulator of follicular growth --we validated that two pathway-associated proteins, tubulin α4a (TUBA4A) and tubulin α 1C (TUBA1C), exhibited expression patterns consistent with proteomic data.

**Discussion:**

Our quantitative multi-omics findings define the molecular landscape underlying follicular size-dependent developmental differences, provide direct insights into the physiological status of follicular development, and offer actionable guidance for optimizing in vitro maturation (IVM) media for immature oocytes.

## Introduction

*In vitro* maturation (IVM) is a kind of assisted reproductive technology (ART). It has attracted more and more attention because of its safety, convenience, low cost, high benefit and and effective reduction of the occurrence of ovarian hyper stimulation syndrome. Reproductive experts believe that it can effectively preserve the fertility of patients with polycystic ovary syndrome, cancer or risk of premature ovarian failure (Sirait et al., 2022). The existing IVM medium is a culture system based on the production of follicular fluid. Follicular fluid is composed of secretions of granulosa cells, oocytes, theca cell in follicles and some plasma proteins in serum. It is a place where oocytes are highly active in growth and metabolism, and plays a vital role in the growth and development of oocytes. Follicular fluid constitutes an important microenvironment for oocyte development, which can indirectly reflect the growth and maturation efficiency and pathophysiological status of oocytes (Gairola et al., 2022). Follicular fluid and its effect on the development of oocytes is a research hotspot in the field of assisted reproduction. A large number of studies have found a positive correlation between follicle size and oocyte development ability in different species. Coculture of follicular fluid from larger follicles with oocytes from small follicles promoted oocyte maturation (Li et al., 2024). Studying the complex composition of follicular fluid is helpful to understand the molecular mechanisms involved in oocyte maturation and embryo development. However, the dynamic changes of proteins and metabolites during folliculogenesis have not been comprehensively analyzed. Yang et al. (2020) conducted metabolome studies on human follicular fluid with a diameter smaller than 13 mm and a diameter larger than 17 mm, and found that the size of follicles affects the metabolic characteristics of human follicular fluid. Dehydroepiandrosterone is negatively correlated with oocyte maturation rate and high-quality embryo rate, which can be used to evaluate the potential development ability of follicles. Additionally, the same research team Yang et al. found that lysophosphatidylcholine (LPC) can serve as a biomarker for follicular development and ovarian sensitivity (Yang et al., 2022). PLA (Chen et al., 2021) studied the proteomics of follicular fluid of follicles with a diameter of about 6.1 mm and found that 24 proteins were related to the process of follicle regulation; 35 and 65 proteins were downregulated and upregulated respectively in the follicular fluid of oocytes that could develop to MII stage. However, the results of combined analysis of proteome and metabolomics of follicular fluid are not clear. Compared with single omics analysis, multi omics integrated analysis of proteomics and metabolomics can more clearly reveal biological mechanisms and molecular characteristics to predict their potential roles in complex biological systems. In this study, Based on the authors’ clinical experience and referencing commonly used thresholds in prior follicular development studies (Li et al., 2022), this study defined a mature follicle group (diameter ≥ 18 mm) and an immature follicle group (diameter < 14 mm). Proteomics and metabolomics were combined to detect the differential expression between follicular fluid groups with different diameter follicles, to find proteins and metabolites that play a key role in follicle growth and development and oocyte maturation, and to enrich our omics information about human follicular fluid. The specific proteins tubulin were identified as biomarkers for oocyte maturation and IVM technology application, in order to establish a more scientific and effective IVM system, improve the efficiency of ART, and provide theoretical support for the development of commercial IVM medium.

## Materials and methods

### Research object

This study was carried out in accordance with relevant guidelines and regulations and was approved by the Ethics Committee of Henan Provincial People’s Hospital (SYSZ-LL-2021091501).All patients and/or their legal guardians provided informed consent. Patients who visited the Reproductive Medicine Center of Henan Provincial People’s Hospital from June 2023 to August 2023 were included. All patients were ≤35 years old, 18.5 kg/m^2^≤BMI≤24.0 kg/m^2^, first cycle in our center, normal ovarian reserve function, The main causes of infertility were tubal factors or male factors. Patients with chromosomal abnormalities, endocrine diseases, uterine diseases, infectious diseases, tobacco and alcohol addiction, and incomplete important information were excluded.

### Ovulation stimulating protocol

All the studies were conducted by the same clinical team according to the patient’s condition, according to the routine selection of individualized ovulation induction protocol and regulation monitoring in our center. The samples in this study were independent and derived from distinct patients. To further minimize inter-individual variability, clinical characteristics—including age, body mass index (BMI), and baseline hormone levels—were rigorously matched between the two patient groups. Covariate adjustment was applied in the statistical analysis to address potential confounding effects. All patients chose antagonist protocol for ovulation induction. Exogenous gonadotropin (Gn) (Gonal-f, Merck, Germany) was administered on the 2nd to 4th day of menstrual cycle to initiate ovulation induction. The starting dose of Gn was selected according to the patient’s age, antral follicle count (AFC), basal sex hormone level, anti-mullerian hormone (AMH) and BMI. The starting dose of Gn was 75–300 IU/d. After 4–5 days of treatment, the dose of Gn and the frequency of follow-up visits were adjusted according to follicle size and hormone levels. When the dominant follicle diameter≥ 14 mm or estrogen (E2) level≥ 600 pg/mL or blood luteinizing hormone (LH) level ≥10 IU/L, gonadotropin-releasing hormone (GnRH) antagonist (Cetrotide, Merck, Germany) (0.25 mg) was added. When the diameter of three dominant follicles≥17 mm or two dominant follicles≥18 mm, combined with the levels of progesterone(P) and E2, 10000IU human chorionic gonadotropin (hCG) (Chorionic Gonadotropin for Injection, Livzon Pharmaceutical Group Inc.,China) was injected. 35.5–37 h later, vaginal ultrasonic-guided oocyte retrieval was performed. *In Vitro* Fertilization-Embryo Transfer/Intracytoplasmic Sperm Injection was selected according to the male’s condition.

### Collection and processing of follicular fluid

Before needle insertion, the diameter of the target follicle was measured and recorded via transvaginal ultrasound. Follicular fluid (FF) free of visible blood or irrigation fluid was then aspirated. Follicles with a diameter >18 mm were assigned to the large follicle group, and those with a diameter <14 mm were assigned to the small follicle group. Follicular fluid samples were analyzed individually to preserve patient-specific information and avoid potential bias introduced by pooling. Immediately after collection, the samples were centrifuged at 2,000 × g for 10 min at 4 °C. The supernatant was aliquoted into sterile tubes and stored at −80 °C until use.

### Protein extraction and protein quality detection

In this part, samples were pretreated using the iST sample pretreatment kit (PreOmics, catalog number (Cat. No.) P.O.00027). The lysis buffer of this kit can accomplish lysis, denaturation, alkylation, and reduction in a single step. Add protease inhibitors into the lysate, vortex and shake to mix well, and centrifuge instantaneously; Take out follicular fluid samples in the frozen state, add pre cooled lysate according to a certain proportion, and shake three times with a high-throughput tissue grinder; Lysis on ice for 30 min; Centrifuge at 12000 *g* for 30 min at 4 °C and collect the supernatant. Dilute the follicular fluid sample, and take 10 uL of follicular fluid sample and standard sample respectively; Add 150 uL BCA working solution (Cat. No. P0010, Beyotime Biotechnology) prepared respectively. Shake well, and react at 37 °C for 30 min; A562 was measured by microplate reader, and the protein concentration of follicular fluid samples was calculated. Electrophoresis; Coomassie brilliant blue staining, decolorization solution decolorization.

### Proteolysis and pretreatment for mass spectrometry

Take 100 μg protein sample, add 8M urea 0.1% SDS lysate to 90 μL, vortex and mix well, add 10 μL TEAB (Cat. No. 40064460, Shanghai Sigma High Technology Co., Ltd), and adjust its pH to 8; After reduction, alkylation and precipitation, restore the sample to 4 °C, centrifuge at 10000 *g* 8 °C for 20 min, and discard the supernatant; Add 90% acetone (Cat. No. 40064460, Traditional Chinese Medicine) pre cooled at −20 °C to wash the protein, vortex and mix well, centrifuge at 10000 *g* 8 °C for 20 min, and discard the supernatant. Wash the sediment repeatedly; Discard the supernatant, put the centrifuge tube into the fume hood to volatilize acetone completely, and add 100 μL 100 mM TEAB to fully dissolve the protein precipitate; Add enzyme to the protein, add 4 μL of 0.5 μg/μL trypsin (Cat. No. V5117, Promega Corporation) after the sample is centrifuged transiently, and mix overnight at 37 °C 500 rpm; The next day, 2 μL of enzyme was added into the reaction tube, and thereaction continued for 2 h. Take out the enzymatically hydrolyzed sample and use the desalting column for desalting; The pierce quantitative colorimetric peptide assay kit (Cat. No. 23275, Thermo Fisher) was used for peptide quantification, and the total amount of peptide was calculated and drained.

### The spectrogram database and DIA data analysis were established

The original data were merged and analyzed by Spectronaut X to search the database, and the default parameters of the software were used to build the database. The database was set up for Trypsin enzymolysis using Uniprot or the database provided. The false positive rate (FDR) was set to 1% at both the parent ion and peptide levels. Each sample was added with 30 μL solvent A to make a suspension, 9 μL was removed and 1 μL of 10×iRT peptide was added. After mixing, the samples were separated by nano-LC and analyzed by online electrospray tandem mass spectrometry. The mass spectrometer adopts the diaPASEF mode for Data - Independent Acquisition (DIA) data collection. The scanning range is from 349 to 1,229 m/z, and the width of the isolation window is set to 40 Da. During the PASEF MS/MS scanning process, the collision energy increases linearly with ion mobility, rising from 59 eV (with 1/K_0_ = 1.6 Vs/cm^2^) to 20 eV (with 1/K_0_ = 0.6 Vs/cm^2^). To analyze the DIA data, the default parameters of Spectronaut 18 (BGS Factory Settings (default)) were adopted. The sequence database was GRCh38.p14 (Ensembl_release110) protein database, and trypsin digestion was set. For the search parameters, fixed modifications included Carbamidomethylation, while variable modifications included Oxidation. The iRT peptide software could automatically correct the retention time and mass window and determine the ideal extraction window. The criteria for protein identification were as follows: Precursor Threshold 1.0% FDR and Protein Threshold 1.0% FDR. The decoy database was generated using the mutated strategy, which was similar to shuffling a random number (at least 2 amino acids and at most half of the total peptide length) of amino acid sequences. Spectronaut performed automatic correction, and a local normalization strategy was used for data normalization. Peptides with less than 1.0% FDR were used for protein group quantification via MaxLFQ. And all MS1 that meet the screening criteria are used to calculate expression. The MaxLFQ algorithm was employed for protein quantification in this study (Software version: Spectronaut 18Parameters: Default parameters BGS Factory Settings), and all qualified peptides meeting the criterion of FDR <1% were comprehensively included in the quantification process.

### Bioinformatics analysis of proteomic data

Differential expression protein screening threshold: The absolute value of the Fold change (FC) was greater than 1.5 times (|log2 (1.5)| ≈ 0.58) and *P* value < 0.05. Differentially abundant proteins with significant differences between groups were screened. Proteome Discoverer software was used for database retrieval. Functional annotation was made for all the identified proteins. Functional annotation and enrichment analysis of differential proteins were performed, while data were visualized. Functional annotation, functional enrichment and cluster analysis were performed for all identified proteins and significantly different proteins.

### Follicular fluid metabolite extraction

Under both POS and NEG ion modes, the correlation between QC samples was greater than 0.998, indicating stable instrument performance during the analysis process. After thawing slowly at 4 °C, follicular fluid was mixed with pre-cooled methanol/acetonitrile/water solution, sonicated at low temperature for 30 min, left at −20 °C for 10 min, centrifuged at 14000 *g* 4 °C for 20 min, and the supernatant was dried under vacuum. For mass spectrometry analysis, 100 μL aqueous acetonitrile solution was added to redissolve, vortexed, and centrifuged at 14000 *g* 4 °C for 15 min. The supernatant was injected and analyzed.

### Chromatography-mass spectrometry analysis and data processing

Samples were separated on an Agilent 1,290 Infinity LC ultra-high performance liquid chromatography system. Sequential analysis of samples was performed in random order. The primary and secondary spectra of the samples were collected by AB Triple TOF 6600 mass spectrometer. Raw data were converted to MzML format by proteowizard, and then the XCMS program was used for peak alignment, retention time correction, and peak area extraction. For the data extracted by XCMS, the integrity of the data was first checked, metabolites with more than 50% missing values in the group were removed and did not participate in the subsequent analysis, the null value KNN was filled, the extreme value was deleted, and finally the total peak area was normalized.

### Bioinformatics analysis of metabolomics data

The *P* value was calculated according to statistical test, and the Variable Importance in the Projection (VIP) value was calculated by OPLS-DA dimensionality reduction method. The screening threshold of differential metabolites was VIP value > 1 and *P* value < 0.05. KEGG and HMDB databases were used for functional annotation, cluster analysis and metabolic pathway enrichment analysis.

### Combined analysis of proteome and metabolome

All differentially abundant proteins and differentially abundant metabolites in this study were mapped to the KEGG pathway database to obtain their connections in metabolic pathways. Pearson correlation coefficients were calculated for the integration of metabolomic and proteomic data. Protein and metabolite pairs are listed in descending order of absolute correlation coefficients.

### ELISA analysis of key cytokines

The collection and preparation of follicular fluid samples for Elisa are the same as above. The levels of TUBA4A and TUBA1C in the large-diameter follicle group and the small-diameter follicle group were detected by enzyme-linked immunosorbent assay (ELISA) kit according to the manufacturer’s instructions (catalog no. KS19847; Shanghai Keshun Biotech, Shanghai, China) (catalog no. KS19843; Shanghai Keshun Biotech, Shanghai, China). Absorbance was measured by photometry using a microplate reader at 450 nm and plotted on a standard curve.

### Statistical analysis

SPSS26.0 software was used to analyze the patient’s age, BMI, endometrial thickness, basal AFC on day 2–3 of menstruation, basal follicle-stimulating hormone (FSH), E2, AMH, total dose and duration of Gn use, FSH, E2, LH, P on trigger day, endometrial thickness on trigger day, and the number of oocytes retrieved. Shapiro-Wilk normality test and Levene test were used to verify data normality and variance homogeneity respectively. Measurement data with normal distribution were expressed as mean ± standard deviation (x ± s) and analyzed by independent sample t-test. Non-normal distribution measurement data were expressed as median (quartile) [M(Q1, Q3)], and Mann-Whitney U test was used. The test level α = 0.05.

## Results

### Comparison of baseline data

**FIGURE 1:**
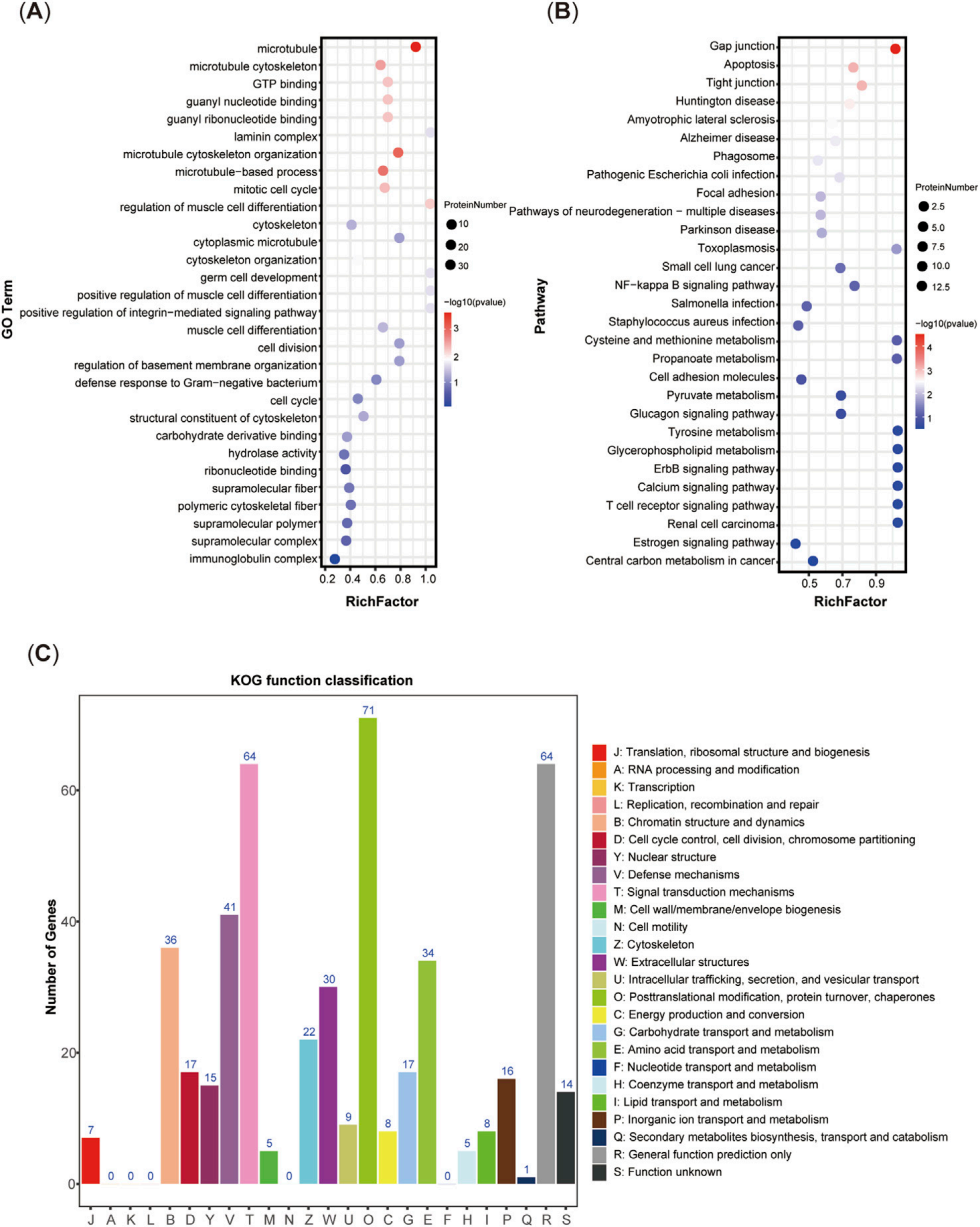
Go enrichment analysis of differentially abundant proteins. **(A)** The color of dots represents the degree of protein enrichment. Red represents high enrichment and blue represents low enrichment. The size of dots represents the number of proteins. The larger the size of the dots, the more the number of proteins. KEGG enrichment analysis of differentially abundant proteins. **(B)** The color of dots represents the degree of protein enrichment. Red represents high enrichment and blue represents low enrichment. The size of dots represents the number of proteins. The larger the size of the dots, the more the number of proteins.KOG functional classification statistics of differentially abundant proteins. **(C)** Different colors represent different pathways. The higher the column, the more protein rich it is.

In this study, follicular fluid samples from 12 patients were equally divided into two groups. As shown in Figure 1, the follicular fluid of follicles with a diameter larger than 18 mm in one group and smaller than 14 mm in the other group. There was no difference in patient age, BMI, endometrial thickness, basal sinus follicle count, basal FSH, basal E2, AMH, total dose and days of Gn use, FSH, E2, LH, P on trigger day, endometrial thickness on trigger day, and number of retrieved eggs (*P* > 0.05). The data are shown in Table 1.

**TABLE 1:** General characteristics of the included patients were collected.

Clinical indicators	<14 mm group (n = 6)	>18 mm group (n = 6)	t/Z	*P* value
Age (year)	32.50 (30.50,33.25)	33.50 (33.00,35.00)	1.917	0.065
BMI (kg/m^2^)	22.22 ± 1.44	20.26 ± 2.03	1.932	0.082
Endometrial thickness (mm)	8.75 ± 1.86	10.20 ± 4.09	−0.791	0.455
AFC (number)	11.50 (10.00,15.75)	12.00 (10.00,14.00)	0.335	0.818
FSH (mIU/mL)	6.52 ± 2.49	8.93 ± 2.30	−1.748	0.111
E2 (pg/mL)	54.97 ± 7.57	50.16 ± 6.75	1.162	0.272
AMH (ng/mL)	2.54 ± 1.22	2.97 ± 1.09	−0.643	0.534
Gn total dose (IU)	1937.50 ± 633.59	2000.00 ± 878.07	−0.141	0.890
Gn days (day)	7.67 ± 1.03	8.33 ± 1.51	−0.894	0.395
HCG dose (IU)	10000 ± 0	10000 ± 0	​	​
FSH on trigger day (mIU/mL)	17.32 ± 6.91	16.80 ± 7.71	0.124	0.904
E2 on trigger day (pg/mL)	1,570.67 ± 635.97	1893.83 ± 508.05	−0.972	0.354
LH on trigger day (mIU/mL)	2.27 ± 1.06	3.39 ± 2.01	−1.208	0.255
P on trigger day (ng/mL)	0.78 (0.36,0.90)	0.55 (0.32,0.92)	−0.564	0.589
Endometrial thickness on	11.33 ± 2.73	11.67 ± 1.21	−0.273	0.790
trigger day (mm)				
Retrieved oocytes (number)	7.17 ± 3.25	7.00 ± 1.79	0.110	0.915

### Identification of proteomic information

We set the filtering standard for protein levels as FDR ≤0.01. The total number of identified spectra was 5305, the number of identified peptides was 4154 and the number of proteins was 551.

### Identification of differentially abundant proteins

A total of 81 differentially abundant proteins were identified in follicular fluid of large and small diameter follicles. The difference threshold is as follows: the absolute value of the FC is greater than 1.5 times (|log2 (1.5)|≈0.58), and *P* value < 0.05. Compared with the small diameter follicle group, 34 proteins were upregulated and 47 proteins were downregulated in the large diameter follicle group.

### Functional classification of differentially abundant proteins

Differentially abundant proteins participate in multiple life processes of the body. GO functional enrichment analysis of differentially abundant proteins showed that proteins were mainly enriched in germ cell development, cell cycle, cell division, microtubule-based processes, cytoskeletal organization, guanylate binding, guanylate ribonucleotide binding, carbohydrate derivative binding, laminin complex, positive regulation of integrin-mediated signaling pathway, supramolecular polymers, hydrolases, basement membrane organization, immunoglobulin complex and other pathways. As shown in Figure 1A.

The results of KEGG enrichment included the top 30 pathways. It revealed apoptosis, E2 signaling pathway, gap junction, tight junction, phagosome, cysteine and methionine metabolism, propionate metabolism, pyruvate metabolism, glucagon signaling pathway, cell adhesion molecules, tyrosine metabolism, glycerophosphatidylcholine metabolism, calcium signaling pathway, T cell receptor signaling pathway, focal adhesion. In addition, some differentially abundant proteins have been detected to be associated with neurodegenerative diseases, such as Huntington’s disease, amyotrophic lateral sclerosis, Alzheimer’s disease, and Parkinson’s disease, as shown in Figure 1B.

In addition, euKaryotic Ortholog Groups (KOG) functional classification statistics of the obtained differentially abundant proteins revealed 71 differentially abundant proteins involved in posttranslational modifications, protein turnover, chaperones. 64 differentially abundant proteins have potential functional roles in signal transduction mechanisms. 36 differentially abundant proteins involved in chromatin structure and dynamics. 17 differentially abundant proteins involved in cell cycle control, cell division, chromosome assignment. In addition, some differentially abundant proteins were also found to play a role in metabolic activities, including amino acid transport and metabolism (34), carbohydrate transport and metabolism (17), inorganic ion transport and metabolism (16), lipid transport and metabolism (8), etc., as shown in Figure 1C.

### Identification of metabolomic data

Metabolomic analysis of human follicular fluid was performed using a non targeted metabolomic strategy. In this study, 10572 metabolites were obtained in positive ion mode and 10841 metabolites were obtained in negative ion mode, including nucleic acids, amino acids, peptides, fatty acids, lipids, carbohydrates, organic acids and other metabolites.

### Determination of differentially abundant metabolites

Differentially abundant metabolites between the two groups were identified according to the VIP>1 and *P* value < 0.05. A total of 141 differentially abundant metabolites were identified between the large-diameter follicle group and the small-diameter follicle group. Among these metabolites, 12 metabolites were upregulated in the large diameter follicle group. However, 129 metabolites were downregulated.

### Bioinformatics analysis of differentially abundant metabolites

**FIGURE 2:**
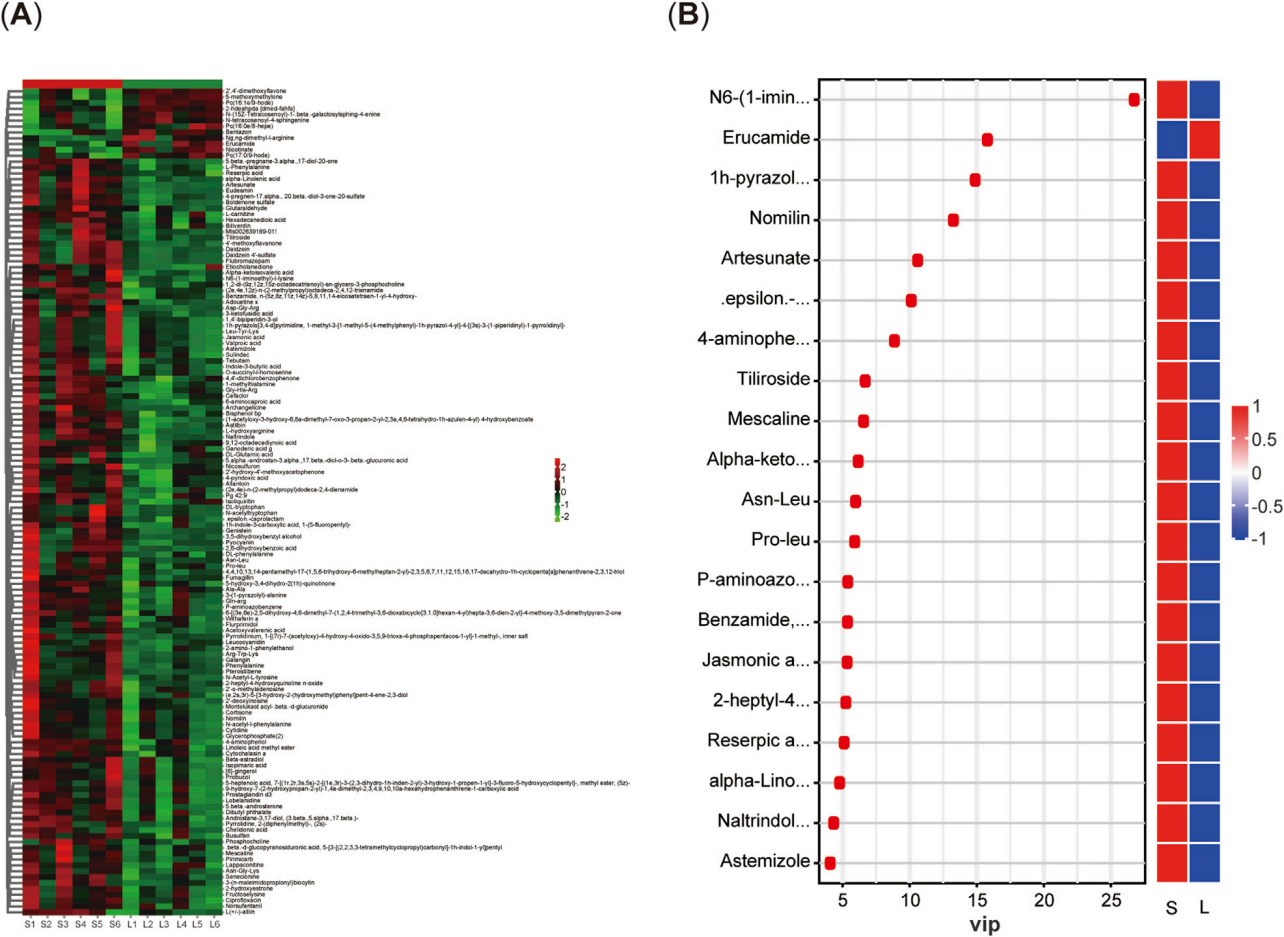
Cluster analysis of differentially abundant metabolites. **(A)** Each column represents a sample, and each row represents a metabolite. Red represents the upregulation of the metabolite expression, green represents the downregulation of the metabolite expression. VIP figure. **(B)** A point in the figure represents a metabolite. The abscissa is the VIP value. The larger the VIP value, the greater the contribution of the substance. The color on the right indicates the abundance of this metabolite in different groups. The red color indicates upregulation, and the blue color indicates downregulation.

The obtained data of differentially abundant metabolites were analyzed by cluster analysis. These two groups of large or small diameter clusters are in opposite patterns. The metabolites enriched in the large-diameter follicle group were less abundant in the small-diameter follicle group, as shown in Figure 2A.

The VIP value provides ranking information on the importance of metabolites, which helps explain the contribution of metabolites to sample classification or prediction. A higher VIP value indicates that the metabolite plays a more important role in sample differences. According to the VIP value, the top 15 differentially abundant metabolites with the largest VIP value were selected. N6-(1-iminoethyl)-l-lysine, 1h-pyrazolo [3,4-d]pyrimidine,1-methyl-3-[1-methyl-5-(4-methylphenyl)-1h-pyrazol-4-yl]-4-[(3S)-3-(1-piperidinyl)-1-pyrimidinyl]-, nomilin, artesunate, Epsilon.-caprolactam, 4-aminophenol, tiliroside, mescaline, alphaketo oisovaleric acid, ASN Leu, pro Leu, p-aminoazobenzene, benzamide, n-(5Z,8Z,11z,14z) −5,8,11,14-eicosatetraen-1-yl-4-hydroxy-, jasmonic acid were enriched in the small diameter follicle group. However, erucamide was more abundant in the large-diameter follicle group, as shown in Figure 2B.

### KEGG metabolic pathway analysis

**FIGURE 3:**
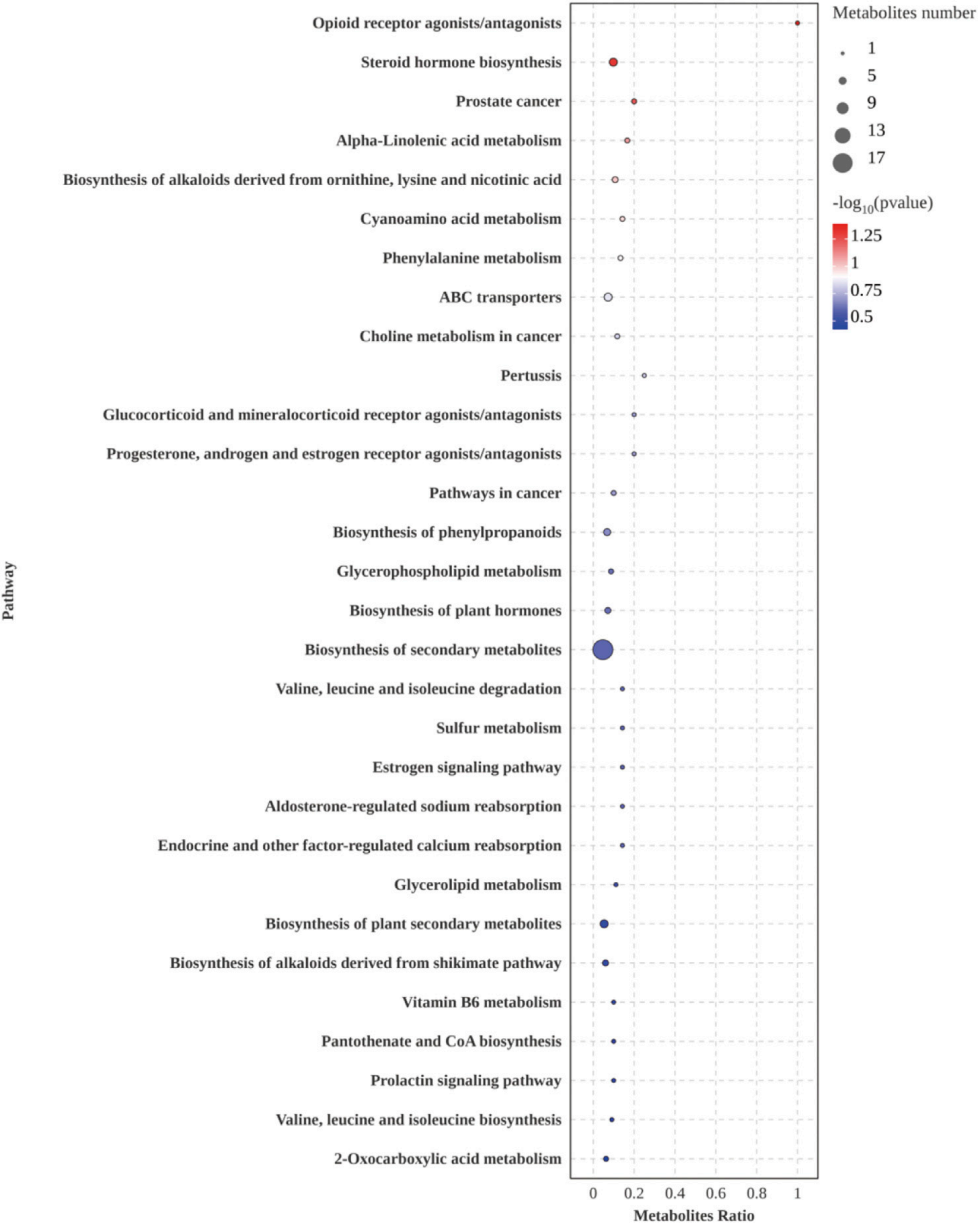
KEGG enrichment analysis of differentially abundant metabolites. The color of dots represents the degree of metabolite enrichment. Red represents high enrichment and blue represents low enrichment. The size of dots represents the number of metabolites. The larger the size of the dots, the more the number of metabolites.

KEGG enrichment analysis of differential metabolites was performed. The top 30 pathways were opioid receptor ligands, steroid hormone biosynthesis, α -linolenic acid metabolism, ornithine, lysine and nicotinic acid alkaloid biosynthesis, cyanoamino acid metabolism, phenylalanine metabolism, ABC transporters, choline metabolism in cancer, glucocorticoid and mineralocorticoid receptor ligands, progesterone androgen and estrogen receptor ligands, and phenylpropanoid Biosynthesis, glycerophospholipid metabolism, biosynthesis of secondary metabolites, valine-leucine and isoleucine degradation, sulfur metabolism, estrogen signaling pathway, aldosterone regulation of sodium reabsorption, endocrine and other factors regulation of calcium reabsorption, triglyceride metabolism, shikimate pathway alkaloid biosynthesis, vitamin B6 metabolism, pantothenic acid and coenzyme A biosynthesis, prolactin signaling pathway. Pathway, valine, leucine and isoleucine biosynthesis, 2-oxo carboxylic acid metabolism, etc., are shown in Figure 3.

### Joint analysis of proteomics and metabolomics

**FIGURE 4:**
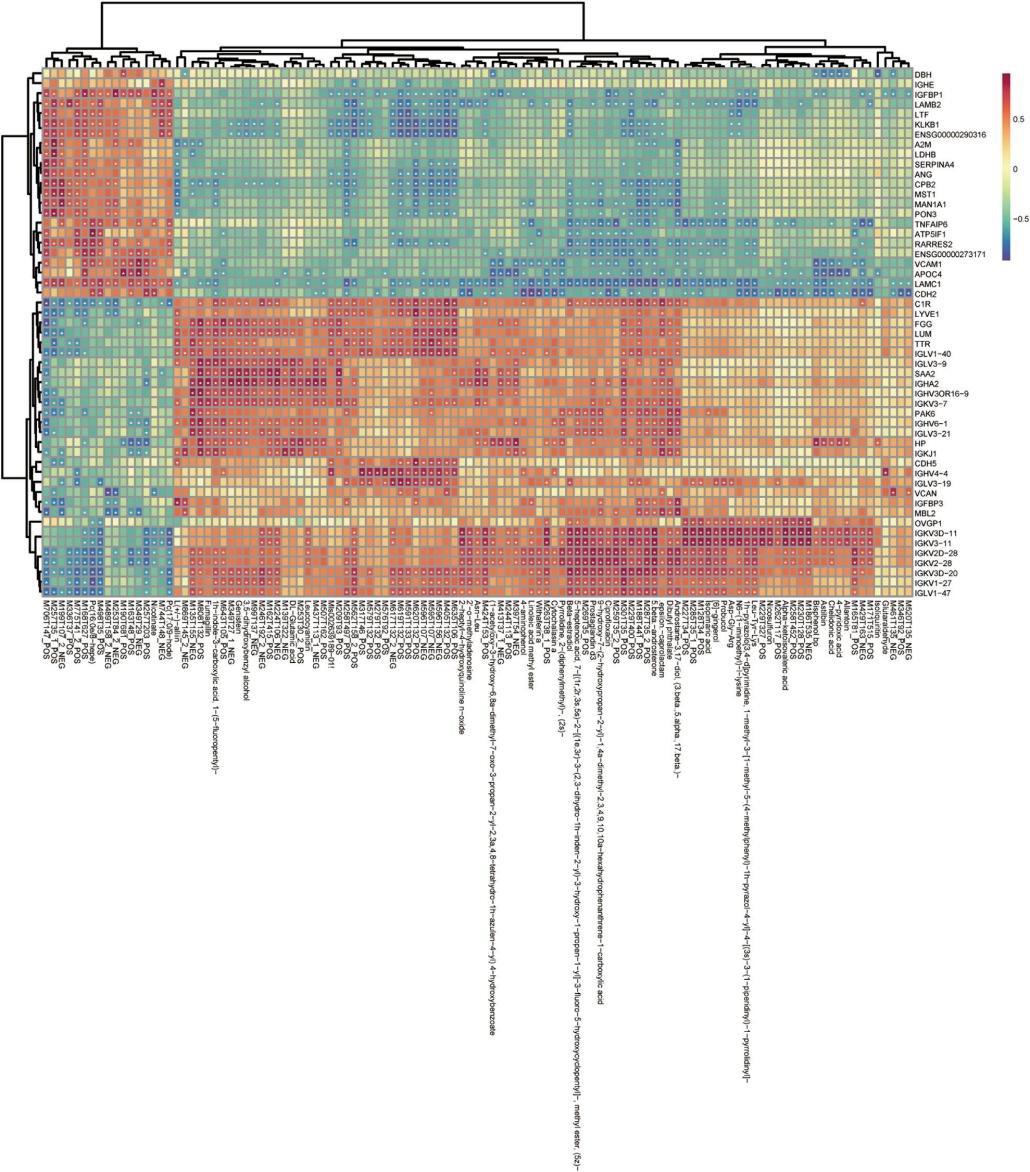
Heat map. The correlation between differential proteins and differential metabolites ranked in the top 250 of the correlation coefficient is shown in a heat map.

Spearman rank sum correlation was used to analyze the correlation between the differentially abundant proteins and differentially abundant metabolites obtained in this study. The top 250 correlated differentially abundant proteins and differentially abundant metabolites are shown in heat maps. This paper provides several typical examples of correlation coefficients higher than 0.9 or less than −0.9. There was a high positive correlation between ATP synthase inhibitory factor subunit 1 and Pc(16:0e/8-hepe), immunoglobulin kappa variable 3D-11 and M186T535_NEG, immunoglobulin kappa variable 3-11 and M186T535_NEG, immunoglobulin heavy variable 3/OR16-9 and M808T132_POS, alpha-2-macroglobulin and M257T35_1_POS, immunoglobulin kappa variable 3D-11 and Alpha-ketoisovaleric acid, immunoglobulin kappa variable 3-11 and Alpha-ketoisovaleric acid apolipoprotein C4 and M349T29_NEG, lactate dehydrogenase B and M257T35_1_POS, immunoglobulin heavy constant alpha 2 and M808T132_POS. There is a strong negative correlation between cadherin 2 and Linoleic acid methyl ester, complement C1r and M706T147_POS, versican and M489T158_2_NEG, as shown in Figure 4.

By analyzing the common pathway of differentially abundant proteins and differentially abundant metabolites between the two groups, It was found that the common differential pathways between the two groups were concentrated in cellular processes (gap junction, apoptosis), metabolism (cysteine and methionine metabolism, propionic acid metabolism), Human diseases (Huntington’s disease, amyotrophic lateral sclerosis, Alzheimer’s disease, Parkinson’s disease, neurodegeneration pathways - a variety of diseases) and other important metabolic processes.

### ELISA validation

**FIGURE 5:**
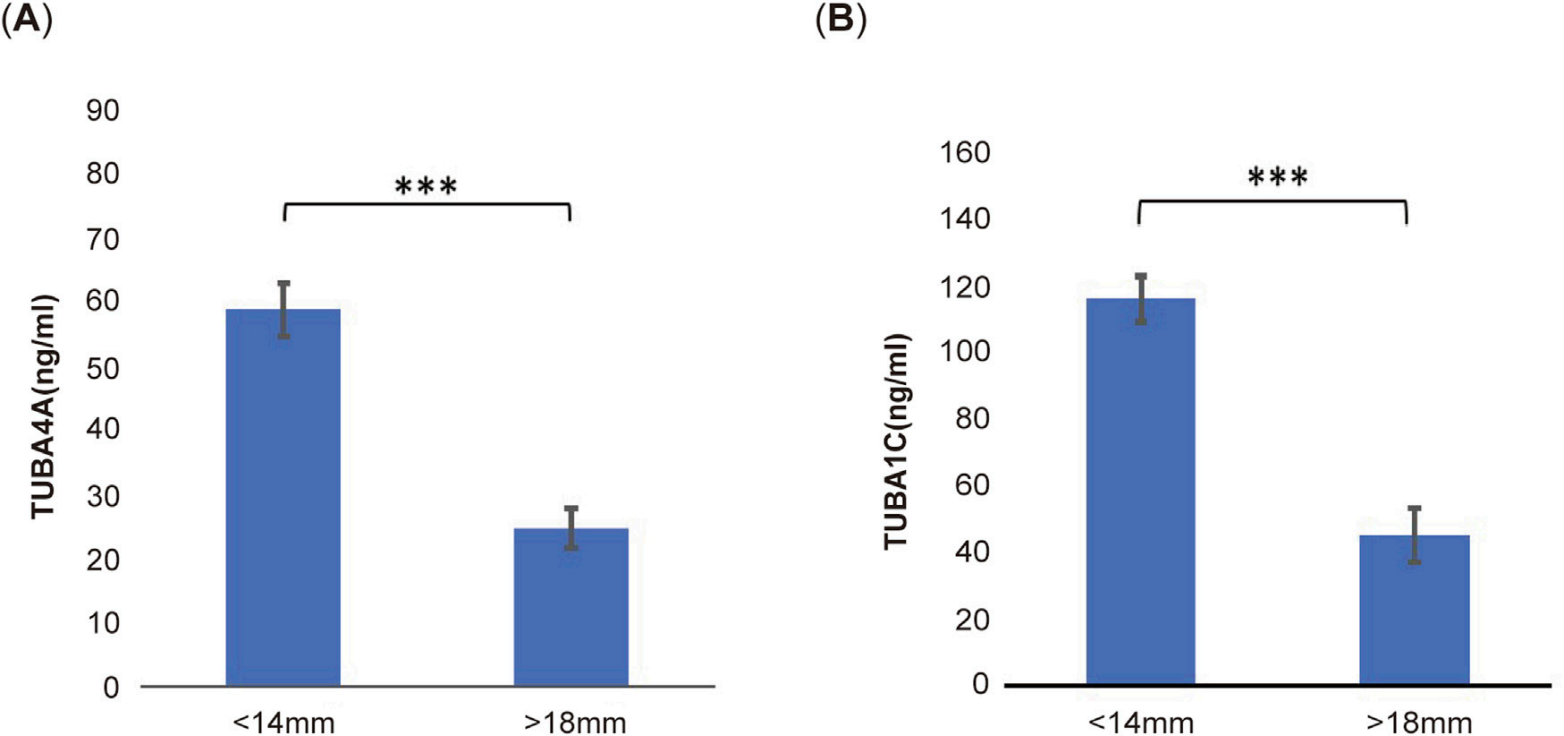
ELISA analysis of TUBA4A. **(A)** The height of the column represents the concentration of TUBA4A of the two groups respectively. “***” represent significantly different (*p* < 0.001). ELISA analysis of TUBA1C. **(B)** The height of the column represents the concentration of TUBA1C of the two groups respectively. “***” represent significantly different of the two groups (*p* < 0.001).

In order to confirm the accuracy of the relevant results obtained, the apoptosis pathway was selected for further study. TUBA4A and TUBA1C, the key proteins in the apoptosis pathway, were selected for ELISA analysis. The results showed that the abundance of these two proteins in the large-diameter follicle group was significantly lower than that in the small-diameter follicle group, and the difference was statistically significant (P < 0.05), which was consistent with the omics results, as shown in Figures 5A,B.

## Discussion

Follicular fluid (FF), a critical component of the female reproductive system, serves as a biochemical conduit mediating communication between the oocyte and its surrounding microenvironment. Analysis of FF enables characterization of the biochemical milieu governing oocyte development; in-depth molecular profiling of FF holds promise for identifying key fertility biomarkers and informing the optimization of *in vitro* maturation (IVM) technology. In this study, we employed multi-omics integrated analysis—combining data-independent acquisition (DIA) proteomics and non-targeted metabolomics—to investigate how protein and metabolite constituents in FF regulate oocyte maturation. Referring to prior studies (Zhou et al., 2022; De Kleijn et al., 2002), we established proteomic and metabolomic datasets from human FF samples of follicles with varying diameters (small sample size), followed by pathway analysis of differentially abundant proteins (DAPs) and differentially abundant metabolites (DAMs). A sample size of 6 cases per group is feasible for the preliminary exploration of differential profiles in follicular development-related research. We acknowledge the limitations of the small sample size and plan to expand the cohort in future validation studies.

Proteomics is different from genomics in that it is not immutable in living organisms. It is dynamically expressed in living organisms with different growth stages, different tissues and organs, and different physiological states. Therefore, the protein environment of follicular fluid before and after oocyte maturation is different. Studying the differences will help to find the mechanism of oocyte growth, development and maturation. Many of the significantly different proteins are closely related to oocyte growth, maturation, division and apoptosis, and protect oocytes and other follicle cells from physical or oxidative damage. Proteins include haptoglobin (HP) has been suggested to play a role in angiogenesis and vascular remodeling (De Kleijn et al., 2002). The molecular weight of HP protein is 85kD, and the gene (GenBank reference sequence:NG_012651.1) is located on the long arm of chromosome 16 (16q22) (Robson et al., 1969). HP is an angiogenic factor, which can improve the function of endothelial progenitor cells in neovascularization, and plays an important role in the proliferation and differentiation of neovascular endothelial cells (Park et al., 2009). In addition, HP limits the interaction between hemoglobin and NO by binding to hemoglobin, reducing the harmful effect of NO on endothelial function, thereby maintaining vascular homeostasis (Di Masi et al., 2020). HP is also a very effective antioxidant, which can directly inhibit linolenic acid and protect low-density lipoprotein from oxidation by binding to Hb, thus preventing tissue damage (Tseng et al., 2004). Lipopolysaccharide binding protein (LBP) is a glycoprotein with a molecular mass of about 55-65kD (Schumann et al., 1990). It belongs to the lipid transfer protein family, including phospholipid transfer protein (PLTP), Cholesterol ester transfer protein (CETP) and bacteria/permeability increasing protein (BPI) (Schumann and Latz, 2000). LBP is a novel PLTP, which differs from other phospholipid transferases in that it requires sCD14 as a soluble carrier protein for optimal phospholipid transfer activity. Under the mediation of LBP and CD14, phosphatidylinositol (PI), phosphatidylcholine (PC) and phosphatidylethanolamine (R-PE) are transported from the membrane with high concentration of phospholipids to reconstituted HDL (rHDL) particles, which is accomplished with the binding of PI and R-PE to sCD14 catalyzed by LBP (Yu et al., 1997). The CETP gene is located on chromosome 16 (16q21-22), which is 25 Kb in length and contains 16 exons and 15 introns (Xue et al., 2023). CETP gene belongs to LBP gene family, and its encoded CETP protein is an important lipid transporter in the process of lipid metabolism (Nurmohamed et al., 2022). CETP transfers cholesteryl esters and triglycerides between HDL and another lipoprotein through a shuttle mechanism or the formation of a bridging ternary complex (Shrestha et al., 2018). Apolipoprotein C4 (APOC4) gene is located in the APOE/C1/C4/C2 gene cluster on chromosome 19 (19q13.32) and is involved in triglyceride metabolism (Pirim et al., 2019). The contents of LBP, CETP and APOC4 in the large follicle group were significantly higher than those in the small follicle group by more than 2 times. It is speculated that LBP and CETP are involved in the transport of lipid metabolites (especially TG), regulate the concentration and structure of HDL, maintain the stability of lipid content in the late stage of oocyte maturation, and play an important role in the composition of lipid in follicular fluid. These differentially abundant proteins, which are closely related to the growth, development and maturation of oocytes in the follicular fluid, may be used as target proteins in IVM culture medium.

During *in vivo* maturation, various metabolites of GCs, cumulus cells, oocytes, *etc.*, are secreted into the follicular fluid to regulate oocyte maturation (Chen et al., 2025). Follicular fluid can systematically reflect the metabolite landscape of oocytes during maturation. However, in the process of maturation *in vitro*, there is a lack of GCs and some substances, which will lead to abnormal metabolite profiles in the culture medium and decline in the quality of oocyte maturation. Therefore, we used metabolomic methods to analyze the follicular fluid samples before and after oocyte maturation, that is, follicular fluid samples from follicles with different diameters. The metabolomic results of this study showed that the differentially abundant metabolites in the follicular fluid of follicles with different diameters included nucleosides, amino acids, peptides, carbohydrates, fatty acids, organic and inorganic compounds, etc. Steroid hormone synthesis is a significantly different metabolic pathway, and the differentially abundant metabolites of this pathway include 2-hydroxyestrone, β-stradiol, 5β-androsterone, etiocholanedione. Follicular growth is achieved through the proliferation of GCs and the formation of follicular fluid. Follicular development involves cell proliferation, apoptosis and angiogenesis in follicles. Various steroid hormones are important regulators in the process of follicular development, differentiation, dominance and atresia (Zhang et al., 2024). 17 β -estradiol and androstenedione (A4) support the *in vitro* growth of bovine oocytes by maintaining the physical connection between oocytes and GCs, and promote the acquisition of meiotic competence in oocytes (Vazakidou et al., 2024). Estrogen increases gene expression, activity and length of telomerase, and also activates mitogenic kinase and PI3K signaling, all of which are essential for follicular development (Ueno et al., 2025; Bayne et al., 2011). Estrogen promotes the proliferation of GCs by acting on NF-κB, stimulates follicle angiogenesis by acting on endothelial cells, and then promotes follicle development (Chou and Chen, 2018). Androgen has been proved to stimulate early follicular development by up-regulating androgen receptor (AR) gene (Lissaman et al., 2023) and Ki-67 in GCs to promote GCs proliferation and reduce GCs apoptosis. In addition, AR gene expression is positively correlated with ovarian follicle growth and GC proliferation, and negatively correlated with follicular atresia and GCs apoptosis (Vendola et al., 1998).

Through the comprehensive analysis of proteomics and metabolomics results, we found that the expression of two proteins (TUBA4A、TUBA1C) in follicular fluid was upregulated in small-diameter follicles, thus affecting the apoptosis pathway. We selected TUBA4A and TUBA1C as candidate proteins and verified them by ELISA. TUBA4A and TUBA1C are guanine nucleotide binding proteins, which belong to α-tubulin molecules and are components of microtubules (Janke and Magiera, 2020). The division of eukaryotic cells depends on the spindle, which is a complex and highly dynamic assembly of microtubules that ensures proper segregation of genetic material into the 2 cells (Redemann et al., 2017). Therefore, TUBA4A and TUBA1C are closely related to cell mitosis and meiosis. Cell division depends on the precise spatiotemporal distribution of tyrosinated and detyrosinated microtubules. Detyrosination involves reversible removal of terminal tyrosines of TUBA4A and TUBA1C. Most α-tubulin isoform genes encode a C-terminal tyrosine, which is catalytically removed and ligated as part of the detyrosine/tyrosine cycle (McKenna et al., 2023). Specifically, the centromeric motor CENP-E guides chromosome arrangement in a detyrosine dependent manner during mitosis. CENP-E can “read” the detyrosine of spindle microtubules, and depyrosinated microtubules enhance the motility of CENP-E (Barisic et al., 2015), while tyramine enriched on stellate microtubules is important for the formation and localization of mitotic spindles (Wang et al., 2025). CDC42 signaling in the cell cortex leads to the asymmetry of meiotic spindles and more decaseinization on one side of the egg, leading to the deviation of centromere orientation and the asymmetric distribution of genetic elements (Akera et al., 2017). TUBA4A is highly expressed in human oocytes and weakly expressed in other somatic tissues. All *de novo* mutations and most sporadic mutations in TUBA4A lead to significant instability of microtubule in different extent. TUBA4A plays an important role in human oocyte maturation and early embryonic development (Li et al., 2023). In addition, TUBA1C is involved in oocyte organelle movement (You et al., 2012). Hu et al. identified TUBA1C as a new genetic marker, expanding the genetic and phenotypic spectrum of TUBA4A in female infertility and recurrent preimplantation embryo arrest, and emphasizing the importance of α-tubulin isotype in preimplantation embryo development (Hu et al., 2024). In fresh oocytes, thick and thin microfilaments are distributed along the oocyte cortex, on the contrary, the thick microfilament domain of the plasma membrane is destroyed or lost in aging oocytes (Somfai et al., 2022). Disruption of tubulin polymerization leads to G2/M phase cell cycle arrest and apoptosis (Nagireddy et al., 2022). TUBA4A and TUBA1C playe an important role in the early stage of oocyte development, which was consistent with the sequencing results that TUBA4A and TUBA1C were higher in the small-diameter follicle group than in the large-diameter follicle group. Therefore, it is presumed that TUBA4A and TUBA1C maintain cell structure, regulate cell function, control cell growth and division, and arrest cell apoptosis in the early stage of oocyte maturation.

In summary, we determined proteins and metabolites that may be involved in regulating oocyte maturation by proteomic and metabolomic analysis of follicular fluid from follicles of different diameters. There were 34 upregulated proteins and 47 downregulated proteins in the large diameter follicle group. The identified differentially abundant proteins are mainly involved in important functional pathways of oocytes, such as substance binding, intercellular signaling, cell cycle, cell division, oocyte development, and apoptosis. A total of 141 differentially abundant metabolites were identified, and 129 metabolites were downregulated in the large diameter follicle group, and the differentially abundant metabolites were mainly involved in metabolism and synthetic activity. From the molecular point of view, we have a preliminary understanding of the characteristics of follicular fluid. These findings not only provide basic data support for the clinical application of IVM, but also guide the personalized diagnosis and treatment of infertility patients. We propose that TUBA4A and TUBA1C, identified in this study, could be incorporated into the development of IVM (*In Vitro* Maturation) media and are promising candidates to be added as regulatory factors to act on the *in vitro* maturation of immature oocytes. Additionally, many identified metabolites could serve as supplementary components to optimize steroidogenesis during follicular development. Our team will subsequently conduct *in vitro* validation and dose-response experiments for the relevant components to further clarify their application value. However, the number and types of clinical samples in this study are limited, and more follicular fluid samples or human oocytes with different diameters need to be analyzed by omics to clarify our conclusion.

## Data Availability

Datasets are available on request: The raw data supporting the conclusions of this article will be made available by the authors, without undue reservation (Shujun Yang, yangsj9966@163.com).
